# Towards Semantic Brain Mapping Methodology Based on a Multidimensional Markup of Continuous Russian-Language Texts: an Attempt at Validation and Development

**DOI:** 10.17691/stm2020.12.2.02

**Published:** 2020

**Authors:** B.M. Velichkovsky, V.I. Zabotkina, Z.A. Nosovets, A.A. Kotov, L.Ya. Zaidelman, S.I. Kartashov, A.N. Korosteleva, D.G. Malakhov, V.A. Orlov, A.A. Zinina, E. Goldberg, V.L. Ushakov

**Affiliations:** Professor, Corresponding Member of the Russian Academy of Sciences, Member of Academia Europaea (MAE), Chief Researcher, National Research Center “Kurchatov Institute”, 1 Akademika Kurchatova Square, Moscow, 123182, Russia, , Russian State University for the Humanities, 6 Miusskaya Square, Moscow, 125993, Russia; Senior Professor, Technische Universität Dresden, Zellescher Weg 17, Room A221, Dresden, 01069, Germany;; Professor, Vice-rector, Russian State University for the Humanities, 6 Miusskaya Square, Moscow, 125993, Russia;; Student, National Research Center “Kurchatov Institute”, 1 Akademika Kurchatova Square, Moscow, 123182, Russia;; Leading Researcher, National Research Center “Kurchatov Institute”, 1 Akademika Kurchatova Square, Moscow, 123182, Russia;; Researcher, National Research Center “Kurchatov Institute”, 1 Akademika Kurchatova Square, Moscow, 123182, Russia;; Senior Engineer, National Research Center “Kurchatov Institute”, 1 Akademika Kurchatova Square, Moscow, 123182, Russia;; Researcher, National Research Center “Kurchatov Institute”, 1 Akademika Kurchatova Square, Moscow, 123182, Russia;; Researcher, National Research Center “Kurchatov Institute”, 1 Akademika Kurchatova Square, Moscow, 123182, Russia;; Researcher, National Research Center “Kurchatov Institute”, 1 Akademika Kurchatova Square, Moscow, 123182, Russia;; Researcher, National Research Center “Kurchatov Institute”, 1 Akademika Kurchatova Square, Moscow, 123182, Russia;; Professor, New York University School of Medicine, 550 1^st^ Avenue, New York, NY 10016, USA;; Leading Researcher, National Research Center “Kurchatov Institute”, 1 Akademika Kurchatova Square, Moscow, 123182, Russia; Associate Professor, National Research Nuclear University MEPhI, 31 Kashirskoe Shosse, Moscow, 115409, Russia

**Keywords:** ecological validity, BOLD signal, modeling in semantics, principal components analysis, cognitive subtraction, haemodynamic response function, subcortical structures, hemispheric lateralization

## Abstract

In the present study, we combine linguistic annotation of oral texts in Russian with the registration of BOLD signal in functional MRI experiments to determine how and where semantic categories are represented in the human brain. Using the same stimuli material, we also analyze the differences in cortical activation in three thematic domains: description of nature, description of working principles of technical devices and more self-referential texts, addressing the question of human identity in conflict situations. We discuss methodological problems within the two approaches (microanalysis and macroanalysis) to study brain activation in natural conditions, i.e. under a continuous speech flow. Within the thematic domain studies, only minimally significant differences in brain activation were registered during the listening to texts from the three thematic groups. This outcome leads to the conclusion that the approach of thematic group contrasts (cognitive subtraction methodology) is not sufficient to study the mechanisms of text comprehension, and should be replaced by the modeling of multidimensional representations of semantic categories in time. Within the semantic category approach, we describe the neurolinguistic process of text understanding as the activation of 15 clusters responsible for semantic categories (e.g. *“Conflict”, “Mental”, “Social”*). Our data demonstrate that the clusters are widely distributed across the human brain. In contrast to the previous studies, we suggest that deep subcortical structures are involved in the processing of certain categories as well. The observed lateralization of category processing underlines the involvement of the right hemisphere in the processing of meaning.

Ask not what is inside of your head, but what your head is inside of.

*James J. Gibson*

Non-invasive brain imaging techniques such as functional magnetic resonance imaging (fMRI) have established an impressive link between psychological investigation of cognitive functions and neuroscience. However, the ecological validity of these new converging studies usually is rather low. This can be the major weakness of cognitive neuroscience as the findings may change dramatically when, for instance, in memory research simple alpha-numerical stimuli are replaced with complex natural material [1–3].

Similarly, the bulk of results from psycholinguistic and neurolinguistic studies could be artifacts of the artificial character of tasks and stimulus material, because the majority of these studies have been conducted with isolated words or, at best, with single sentences. Although semantic selectivity of some brain areas has been known from clinical observations for decades [4], it was owing to the seminal work by Huth and colleagues [5] that the problem of brain’s semantic selectivity was attempted to be solved in a general way. These authors systematically identified semantic selectivity for English in 7 native speakers across the cortex using voxel-wise modeling of fMRI data collected during subjects listening to hours of meaningful narrative stories. Due to complex multidimensional computations with several rather arbitrary steps of reducing uncertainty used in the modeling, many questions remained even years after the study. Until now there is no replication of Huth’s et al. results from other research groups, or for other languages.

We recently started a similar line of research with continuous fragments of Russian spoken language using a more traditional cognitive subtraction methodology [6]; however, we were unable to demonstrate a stable semantic mapping. Several reasons may have contributed to this failure, both conceptual and technical. First of all, it could be the general limitations of Donders cognitive subtraction methodology in neurocognitive research [7, 8]. Secondly, there have been flaws in our stimulus material selection: in difference to that of Huth et al. [5], it was not self-referential and not emotional. Finally, we used in these earlier experiments the standard fMRI scanning protocol with repetition time (RT) of 2000 ms, which may have been too slow for measuring the speech flow.

In the present study, we have attempted to correct the limitations of our earlier experiments. We also attempted to replicate and expand the approach used by Huth et al. [5]. Accordingly, we employed two data-processing approaches. The first approach (macroanalysis) consisted of contrasting general brain activation effects of three radically different groups of texts, one of which now described personal episodes of life with elements of threat and its resolution, while two other were descriptions of nature and the working principles of technical devices. In the second, more in-depth approach (microanalyses), we moved closer to the original Huth’s methodology. In this microanalyses, we took into account the typical multidimensional contexts of a particular word use in the Russian language. We also generally improved the temporal resolution of our BOLD signal measurement by introducing new protocol of ultrafast multiband scanning.

## Materials and Methods

***Text material and its markup annotation.*** As a stimulus material, we have selected and partially produced anew 15 short texts (about 150 words) in Russian, divided into three thematic groups. The first theme was the beauty of nature: five fragments from the works of famous Russian writers (Konstantin Paustovsky, Ivan Turgenev, and others) with description of nature. These highly literary texts describe forests, the sky, plants, and birds without mentioning actions or events. The second theme was the working of technical devices: five texts, each of which describes the working principle of a technical device such as steam engine or cylinder door lock. Texts of this group were rather instructional, written in simple language and not containing technical subtleties. Finally, the third group of texts was about partially dramatic circumstances of contemporary life: five stories were first-person narratives describing a short emotional story experienced by one of the authors of this study.

All the texts were audio recorded by a professional broadcasting speaker (male). We also provided the texts with linguistic markup according to the following sequential algorithm: (a) time annotation, (b) lemmatization, (c) vectorization, (d) feature words annotation. We describe each step of the markup production below.

(a) Time annotation has been assigned in ELAN annotation software: for each word, its temporal boundaries in audio recording were manually defined. The annotation has been double-checked by two experts.

(b) Each word has been annotated by its lexeme (written — write, things — thing) in order to reduce the rich morphological inflections in Russian. This was performed with the help of pymorphy2 software [9]. Pymorphy does not take into account word contexts and therefore cannot guarantee an automatic selection of a correct variant in case of homonymy: it suggests a list of possible variants ordered by the decreasing probability.

In order to select the correct lexeme, the automatic lemmatization was checked by an expert-linguist, who has corrected the errors.

**Figure 1:**
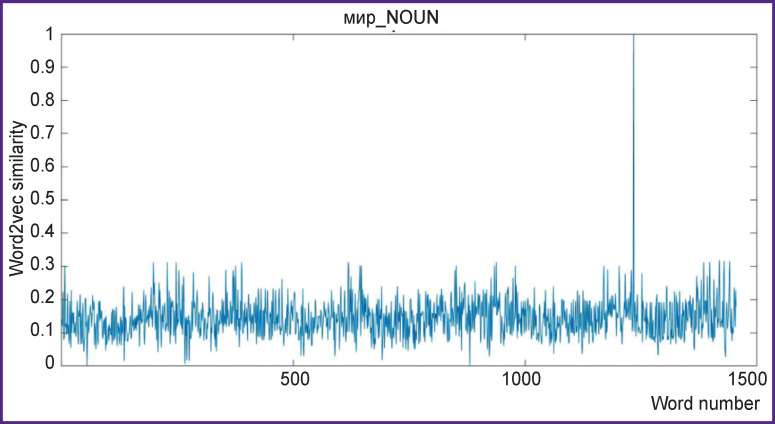
An example of similarity function between words of stimuli texts with the feature word *peace* (мир_NOUN feature) The word2vec similarity value reaches 1 at some time point, where the actual word *peace* appears in text

(c) At the next step of semantic markup, each word was annotated by a semantic vector (word embedding), produced by word2vec method [10], automatically extracted based on the joint occurrence of words in the Russian National Corpus and Russian Wikipedia within the RusVectōrēs project [11].

(d) Words were annotated by vectors of feature words. Semantic distance of words in word2vec space is calculated as a cosine similarity between 300-dimensional vector representations of the words. Since semantically close words have similar contexts, the vector parameters characterizing these words must be close as well (Figure 1 and Table 1). However, this approach to the description of word meanings needs to be extended in order to apply to brain semantic mapping. Therefore, we have selected a list of feature words including 498 most frequent nouns and 499 most frequent verbs [12], which have formed a markup vector^1^. For each of 2241 input text words we have formed a 997-dimensional markup vector, basing on the word2vec cosine similarity between the input word and each of the feature words. As a result, the markup of input words from the texts is a set of 997 numbers, each of which describes the similarity of this word to the feature word. The annotation scheme for the input texts has been combined as a matrix of feature vectors for the text words in time, i.e. assigned to the time of each word appearance in the audio texts.

**Table 1:** An example of stimuli matrix for the group of texts on beauty of nature

0	0	0	год_NOUN	человек_NOUN	время_NOUN	дело_NOUN
фрагмент	00:00.7	00:01.2	0.1328190325780939	0.11861247487522375	0.19820582332505354	-0.005950817967533606
номер	00:01.2	00:01.5	0.20096777926788945	0.1098714356539846	0.1625228053384632	0.14986479297567268
погода	00:03.3	00:03.7	0.10855503527139632	0.1756754253004562	0.2354419469616913	0.13155267699851247
прекрасный	00:03.7	00:04.5	0.1181102934703333	0.18693504656779736	0.19092035271832086	0.18550002163989554
кротко	00:04.8	00:05.1	0.08547306201009564	0.23637058401137523	0.13855906921187044	0.23094816766937054
синеть	00:05.1	00:05.5	0.06550760254245436	0.12605742279988286	0.20414586000302626	0.17294080806225187
майский	00:05.5	00:06.0	0.1866071701558042	0.1321050267349999	0.13487410762369878	0.09455410487650595
небо	00:06.0	00:06.5	0.1331551314405427	0.171750708886239	0.20698841068805285	0.13987553879275083
гладкий	00:07.0	00:07.3	0.0667621308256342	0.14617505963931876	0.17527466033743988	0.13102371193783324
молодой	00:07.3	00:07.7	0.18418924040744622	0.27442796326297647	0.1818189877825005	0.18680062641221995
лист	00:07.7	00:08.0	0.12557060322306124	0.18779267023834567	0.15755408719163222	0.18701390470205315
ракита	00:08.0	00:08.3	0.05574846372403708	0.19466976119073354	0.1256331034429421	0.09493372199985683
блестеть	00:08.3	00:08.7	0.07084179921122535	0.15655404041179888	0.16804858159695157	0.19249045789358232
словно	00:08.7	00:09.1	0.10020265118045335	0.249745808986634	0.2580370715046957	0.15806505957008254
вымыть	00:09.1	00:09.7	0.0815241510217537	0.1263775686837575	0.15492160307081937	0.19508176524147502
широкий	00:10.2	00:10.9	0.10594916942367805	0.1647916042932266	0.16168563359950794	0.14226334380690453
ровный	00:10.9	00:11.3	0.06370461145739958	0.18019710669925748	0.16052564764078503	0.0824265335978116
дорога	00:11.3	00:11.8	0.1723350719087533	0.1891235575333431	0.20619689618486192	0.21206327719861784
весь	00:11.8	00:11.9	0.21871635557442393	0.3731199177299982	0.24923202609127804	0.19078694950456665
покрыть	00:11.9	00:12.3	0.09496991547271749	0.16545026063787427	0.20967727663911617	0.1335365078566172
мелкий	00:12.5	00:12.9	0.0893103058755439	0.19950106144050417	0.14477340509622483	0.20068427248019705
трава	00:12.9	00:13.2	0.11609895995336628	0.14081091753789865	0.16129889207645776	0.20799573436402585
красноватый	00:13.3	00:13.8	0.09567010898411621	0.12653559279953142	0.18299517039386115	0.10372799988421944
стебелек	00:13.8	00:14.5	0.055361991552227874	0.1722707234799712	0.13959579904104202	0.16968889318893443

***Subjects.*** Twenty-five subjects (native speakers of Russian, students of linguistics at the Russian State University for the Humanities, 21*–*28 years old, right-handed and without known history of neurological diseases, 17 females among them) participated in the study. Informed consent was obtained from each participant prior to the experiment. Ethical approval for this study was provided by the local Ethics Committee of the National Research Center “Kurchatov Institute”. All participants were asked to maintain wakefulness with closed eyes during the study.

For the purpose of the experiment, it was important to be sure that a participant really listens to the stimulus material. We controlled this by analyzing corresponding activity of auditory areas of subjects’ brains. In addition, we have prepared one control question for each thematic block of stimulus material. After the test when subject listened to all five texts of one of the topics, the experimenter asked him/her a control question; the subject gave the answer out loud. After the experiment, the subjects filled out a questionnaire, which included a question about their confidence in the accuracy of answers to the questions asked during the experiment. Based on the answers about the texts and the self-confidence assessment, we concluded whether the subject was sufficiently immersed in the perception of texts. Scanning data of four subjects who gave wrong answers to the control questions were excluded from further analysis.

***Design of experiment.*** Counter-balanced block-design was used where the order of presentation of each group of thematically different texts was systematically changed according to Latin square scheme. Within a block the order of stimulus texts was randomized.

***Scanning parameters and pre-processing of BOLD signal.*** Each subject was placed to MAGNETOM Verio 3T (Siemens, Germany) MRI scanner with 32-channel MRI head coil. Structural MRI and resting state BOLD activity were registered individually with closed eyes preceding each experiment. During the experiment, we recorded fMRI data by using ultrafast Multi-band Accelerated EPI Pulse Sequence protocol^2^. The scanning process had two stages: capturing high-resolution anatomical data and recording functional data by a parallel scanning protocol with ultrafast EPI-sequence (TR=1010 ms, TE=33 ms, 56 slices, slice thickness — 2 mm, spatial resolution in each slice — 2×2 mm). Functional data were collected for the resting state condition first (about 8 min) and then for condition of stimulus texts presentation (about 20 min).

BOLD data for each subject were preprocessed using SPM8 software (Wellcome Trust Centre for Neuroimaging, London, UK) in MATLAB R2018a (Mathworks, Natick, USA). Preprocessing consisted of realignment to correct for subject movements, co-registration to align all functional data to subject’s anatomical volume, normalization to convert all images to Montreal Neurological Institute (MNI) space and spatial smoothing with a Gaussian kernel of 8 mm (full width at half maximum).

**Figure 2:**
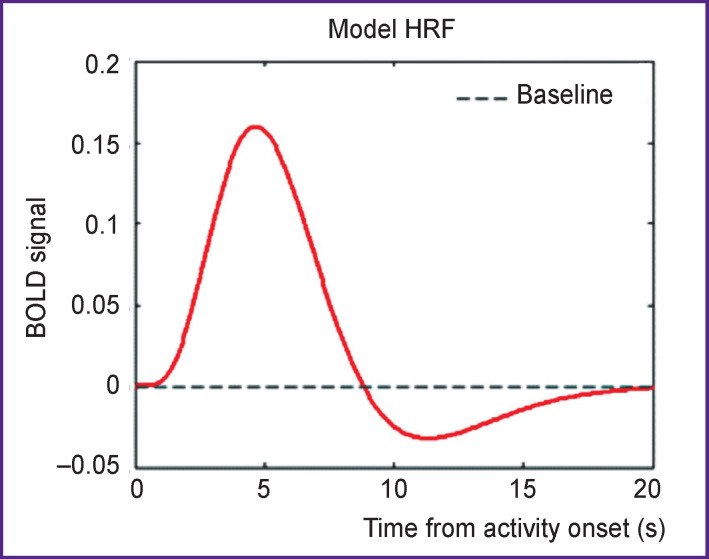
Haemodynamic response function (HRF)

Differences in the BOLD responses evoked by each thematic group of texts were investigated by modeling their associated haemodynamic responses. At the single-subject level, a model was defined using both the onsets and the durations of texts of three categories corrected for a typical delay of the BOLD haemodynamic response function (HRF) shown in Figure 2. These models were estimated in SPM8 (Restricted Maximum Likelihood estimation) using the informed basis set represented by HRF amplitude, derivative and dispersion [12, 13].

In the final reconstruction of brain structures involved in semantic processing, we used individual resting-state data as baseline. For microanalysis of semantic brain mapping for continuous texts several additional processing steps were required. These steps are described below.

***Pre-processing of word representations.*** The stimuli were represented as words, aligned to time of their presentation with the annotation vectors: each word from the text corresponds to a 997 feature vector (see Table 1). Thus, we formed the stimuli matrix [Features  ×  Time samples (Words)], where stimuli are represented as a variation of each feature value over time. An array of time samples was also generated. The Start Time and End Time columns values were converted to seconds and the average values were found. The standard scores (*z*-scores) for the stimuli matrix rows were calculated. These scores are dimensionless quantities allowing us to compare them with the BOLD signal (for which *z*-scores were also found). **The standard ** score calculation formula: *z*=(*x*–x )/*S_x_*, where x is the mean value, *S_x_* is the standard deviation.

**Figure 3:**
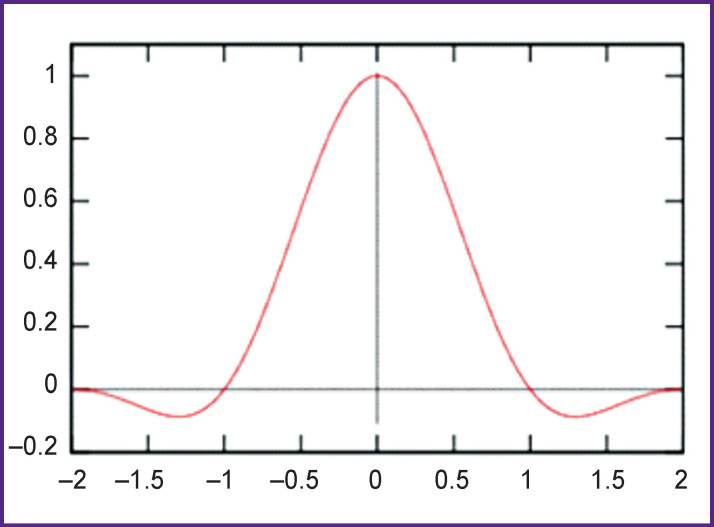
Lánczos Kornél function — see https://en.wikipedia.org/wiki/Lanczos_resampling

For further calculations, it was necessary to bring the time series of semantic vectors in correspondence to the fMRI time scale (with a repetition time of TR=1.1 s). For such a resampling, the Lánczos filter was used (Figure 3) with a cut-off frequency set to the Nyquist frequency of the fMRI acquisition.

**Figure 4:**
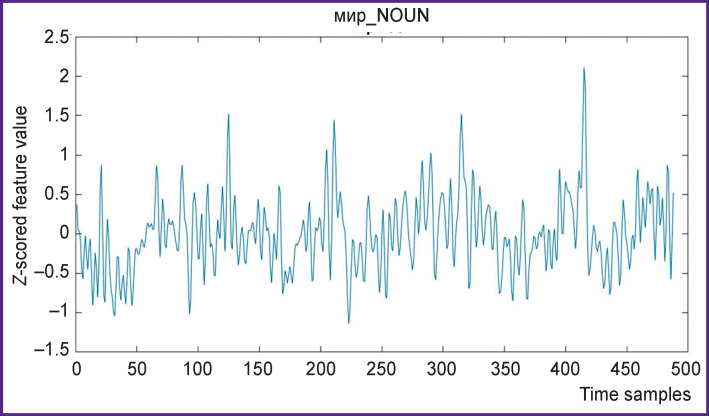
The word *peace* (мир_NOUN) feature time series

**Figure 5:**
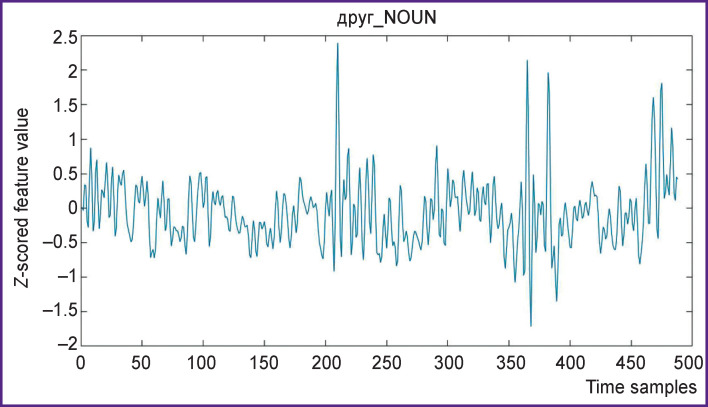
The word *friend* (друг_NOUN) feature time series This word is more common in the text than the мир_NOUN, hence the difference in these graphs to the previous ones (see Figure 4)

Overall 490 samples were received after resampling (Figures 4 and 5), which corresponds to the time series of fMRI data acquisition and to 16 min of stimuli presentation.

In the final step of this pre-processing, the stimuli matrix was considered with respect to typical time delays of the fMRI scanning procedure. The BOLD signal increases and decreases in accordance with the HRF graph. To approximate this curve, 4 point delays were used: 2, 4, 6, and 8 s. Accordingly, 4 copies of time series of each feature were created with these delays and concatenated. As a result, 3988 features were received for each time sample.

**Figure 6:**
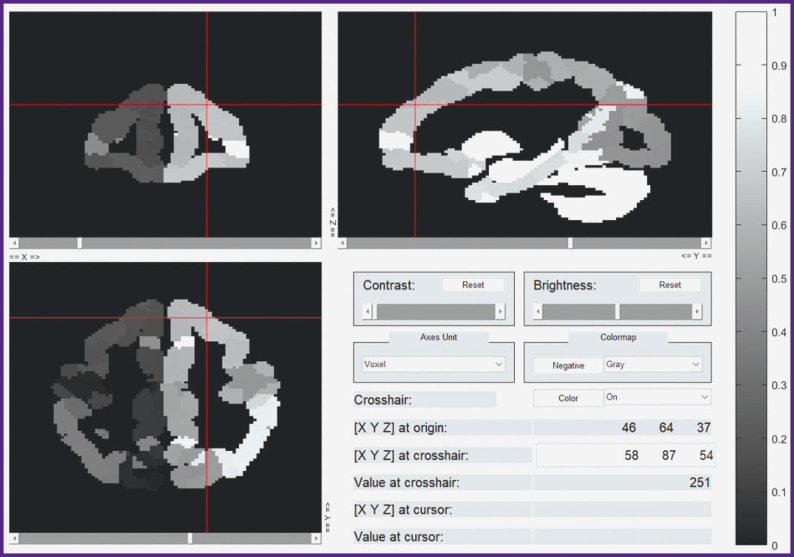
The brain mask representation used in this study

***Estimation of stimulus word to BOLD signal correspondence.*** The next task was to estimate how 997 features affect the BOLD response in each voxel in the cortex and the subcortical structures of the brain. In other words, the task was to predict voxel-wise activation with the highest correlations with the actual data. In order to find these voxels, we applied a specially prepared atlas mask to all the voxels of our data set (Figure 6). The mask represents neocortical gray-matter voxels of both hemispheres, as well as those of some subcortical structures, e.g. amygdalae. After applying the mask only 100,000 voxels (out of 900,000 initially) remained. The time series for these voxels were linearly detrended and *z*-scored in the same way as this was done with the stimuli data.

The following is the description of the regularized linear regression procedure used for the estimation of the weights of each feature for each voxel. Let the *j* voxel time series be *R_jt_*, the *i* semantic feature time series be *S_it_*, and the regression weight of the *i* feature in the *j* voxel be β*_ji_*, *t* — temporal segment, then *R′_jt_*=^Σ^*_i_*b*_ji_S_it_*. To estimate β one usually minimizes squared errors sum:

$$E_{j}(\beta )=\sum_{t}{\left(R_{jt}-R_{jt}^{'}\right)}^{2}=$$

$$=\sum_{t}{\left(R_{jt}-\sum_{i}\beta_{ji}S_{it}\right)}^{2}.$$

This procedure is called the OLS regression, and it does not work directly because the features number (3988) is greater than the time samples number (490). This problem is solved by regularization procedure when the goal is to minimize the following expression:

$$E_{j}(\beta )=\sum_{t}{\left(R_{jt}-\sum_{i}\beta_{ji}S_{it}\right)}^{2}+\alpha \sum_{i}\beta_{ji}^{2}$$

or the same expression in a matrices formula:

$$E=||Y–X\beta |{|}^{2}+\alpha ||\beta |{|}^{2},$$

where *Y* is the BOLD signal matrix (*t*×*m*), *X* is the stimuli matrix (*t*×*p*), α is the regularizing coefficient, β is the weight matrix; *t* is time samples, *m* is the voxel number, *p* is the feature number.

**Figure 7:**
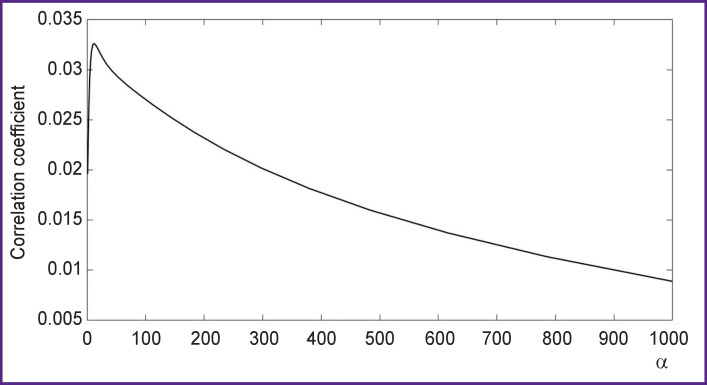
The efficiency curve for α from 1 to 1000

We used the cross-validation method to find the coefficient α. For this purpose, the data set was divided into two parts: in the first part, the weights are estimated for a given α, in the second part these weights are tested. The procedure is repeated for each α of interest. Thereafter α with the best prediction is selected and weights are calculated using the entire dataset and this α. 490-time samples were divided as follows: the first 350 samples were used to construct the weight matrix; the last 140 samples were used to verify the obtained weight matrix by finding the correlation of the BOLD signal time series and the predicted time series obtained by multiplying the stimuli matrix and the weight matrix. In turn, the first 300 of 350 samples were used to find the weights for each α in each voxel, and the last 50 samples were used to select the best α for a given voxel. This procedure was performed for 500 voxels for two sets of α (from 1 to 10 and from 10 to 1000). Correlations were averaged over all voxels for each value of α. The efficiency curves for α from 1 to 1000 were obtained (Figure 7).

**Figure 8:**
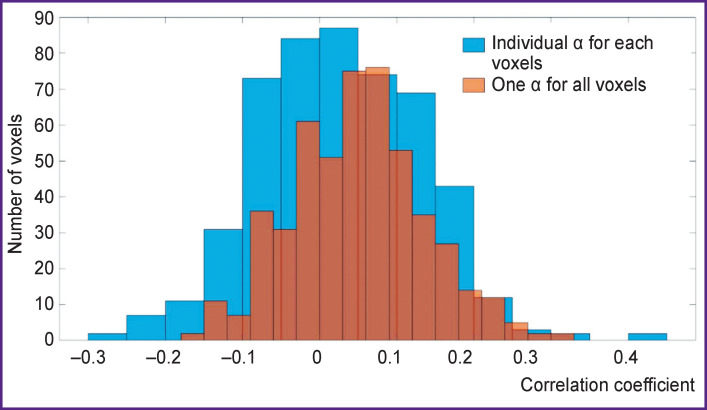
The correlation histogram comparison for different selection of α

As the best, α=12.7427 was chosen because it corresponded to the highest correlation. With this value the weights were calculated on the entire data set. A comparative analysis of the results of the regression with one α for all voxels (obtained by averaging the correlations for all voxels) and the regression results, where each voxel uses its own α (corresponding to the highest correlation of the predicted and presented BOLD signal in a particular voxel), was also performed. Two histograms were made (Figure 8). The graph tells us that one α for all voxels shifts the histogram rightward, relative to 0, so it worked for our purpose better. Therefore, the weights were calculated using the best α=12.7427 for all voxels.

**Figure 9:**
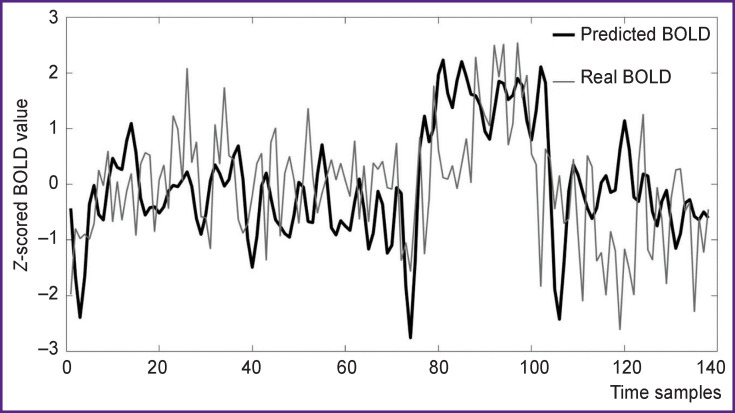
Predicted and real BOLD for correlation coefficient 0.3916

In the next step, predicted (with obtained weights) and real time series were visualized (Figure 9). One can see that the prediction of BOLD activation for correlation coefficient 0.3916 is rather accurate for all these voxels.

***Projecting models onto a lower dimensional subspace.*** In order to find the best voxels to accommodate the semantic features represented by our stimulus words, we have chosen 10,000 voxels with the highest correlation. The principal component analysis (PCA) method was applied to the weight matrices [Voxels  ×  Features]. Before that, the features were averaged over time of haemodynamic responses (that is, out of 3988 features, 997 features were again obtained). For the resulting 10,000 voxels, matrices of scores and loadings were constructed in the principal components space, whereby first four factors of the PCA were used as respective dimensions. After that, we clustered the data with the help of k-means method. To determine the required number of clusters, we have executed a preliminary clustering of lexical semantic vectors (word embeddings) in order to find the clustering depth where clusters represent words from thematic areas (semantic fields), suitable for further combination with BOLD activation data. The number of 15 clusters was selected as the best level of word embedding clustering, which ensured a co-occurrence consistency of words from one semantic field in the same cluster and a minimum of total clusters number.

Following this evaluation, the principal component data for feature words were clustered into 15 clusters via the k-means method. These clusters were subsequently localized in the brain voxels with the MNI coordinates. Since the features define the clusters, it was possible to determine the corresponding voxels. To that end, we utilized the weight matrix, which comprised the features relation to the voxels. Only voxels with the highest weights were used in the following analysis (see the next section).

Can we make this approach more detailed, for example, by defining on an individual level representation of the notion “threat”? To answer the question, we clusterized points in 4D space by method closest neighbor into 12 clusters. For clusterization, we used elements of the space that were most distant from the center with maximal load on the components. This was achieved by repeated finding of 80% random set of points.

The chance of clusterization method and the diminishing clusters’ numbers were caused by the fact that we worked here only with the text from the third group, which has fewer words than the stimulus material as a whole. To find the clusters with threat semantics, we used model vord2vec and build a set of words closest to word *threat*. These were the following words: *danger*, *counter-activity*, *warning*, *insult, worries, reproach, infringement risk, violence*. The distance was computed as median of each word of a cluster to the threatening words. In the end, data were evaluated by an expert.

## Results

To ensure that subjects listened to the stimulus material the activity of auditory brain areas was registered; it showed a satisfactory level of reactivity in all the subjects. Besides this neurophysiological control, we analyzed correctness of subjects’ verbal responses about text contents. The most difficult question was on the texts about nature: “List any bird that was mentioned in the texts you have listened to about nature.” In total, 7 birds’ names appeared in the nature texts. 4 out of 25 participants did not remember any of them and were excluded from the analysis of brain activity. On the question on technical texts, one participant answered incorrectly. The simplest was the question on the texts about life: all the subjects gave the correct answer and only one was not sure of it.

Due to a very high interindividual variability, the results of macroanalysis revealed no systematic group difference in global brain activation among the three groups of texts, either in their direct comparisons or in indirect comparisons relative to the subjects’ resting-state data (t-test Student, SPM). The only significant trend was a higher activation of both amygdalae in the case of perception of texts on working principles of technical devices relative to the other two groups of texts (p<0.005, uncorrected).

A more consistent picture emerged from our microanalysis data. First of all, this is related to the k-means clustering of word features principal component into 15 clusters as described above. The resulting semantic categories are presented in Table 2 with examples of their characteristic key words.

**Table 2:** Clusters of the principal component data for the vectors of 997 feature words

Category	Example features (key words)
Measure (numeric)	*Mass, measure, percent, size, thousand, million, evaluation*
Construction (in space)	*Road, building, window, way, wall, floor, place, space, house*
Social organization	*Government, analysis, attention, data, law, research, method, scientist, error*
Conflict (military)	*War, service, solder, troop, year, period, place, territory, fight, general*
State authority (finances)	*Authority, region, federation, fight, government, state, income, land, population, ruble, republic*
Temporal (procedures)	*Development, solution, participation, method, minute, company, bank, participant, usage, term*
Mental (abstract)	*Knowledge, science, experience, theory, mind, soul, idea*
Corporate (professional)	*Document, boss, employer, director, process, work, telephone, cabinet*
Male (medical, spiritual)	*God, pain, doctor, health, old man, disease, trust, uncle, boy, father, child, parent, body, church*
Female (family)	*Sister, mother, daughter, granny, girl, childhood, wife, family*
History (fate)	*Blood, destiny, fate, history, peace, right, revolution, country, value*
Literature	*Poems, reader, cause, painter, writer, student, captain*
Body parts	*Hair, breast, leg, shoulder, skin, finger, ear, lip, tooth, mouth, heart, tongue*
Industry (production)	*Car, model, technology, factory, production, type, business, order, worker, technology, goods*
Location (home, city)	*Kitchen, room, shop, market, theater, door, hall, corridor, circle, class, stage, frame*

**Figure 10:**
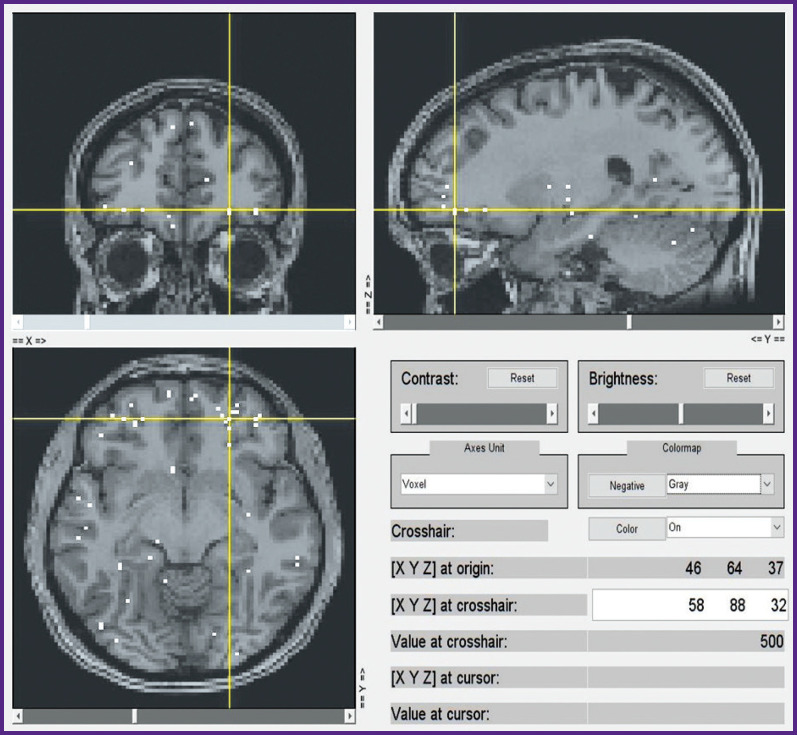
Localization of cluster *“Conflict”*

**Figure 11:**
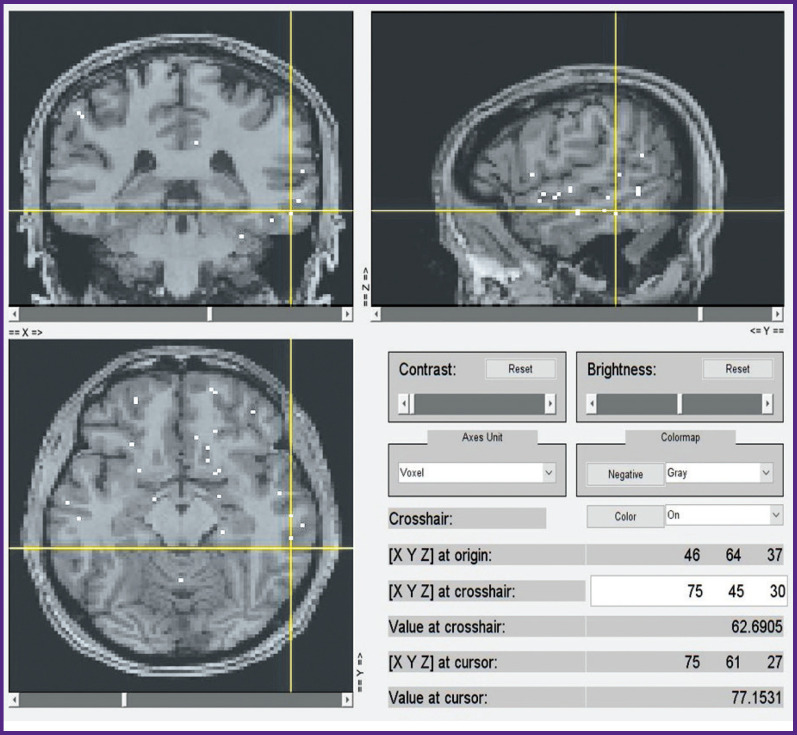
Localization of cluster *“Mental”*

**Figure 12:**
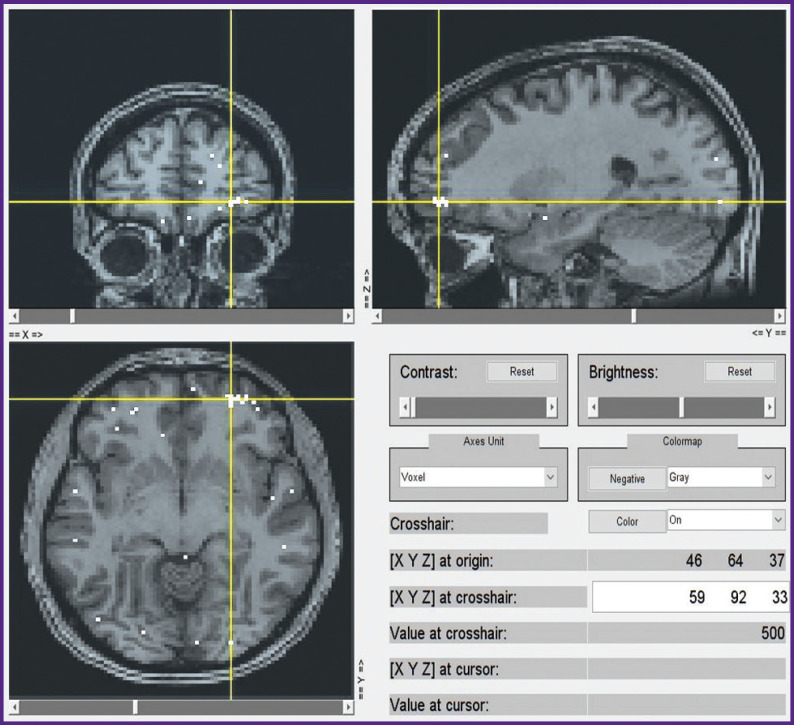
Localization of cluster *“Measure”*

**Figure 13:**
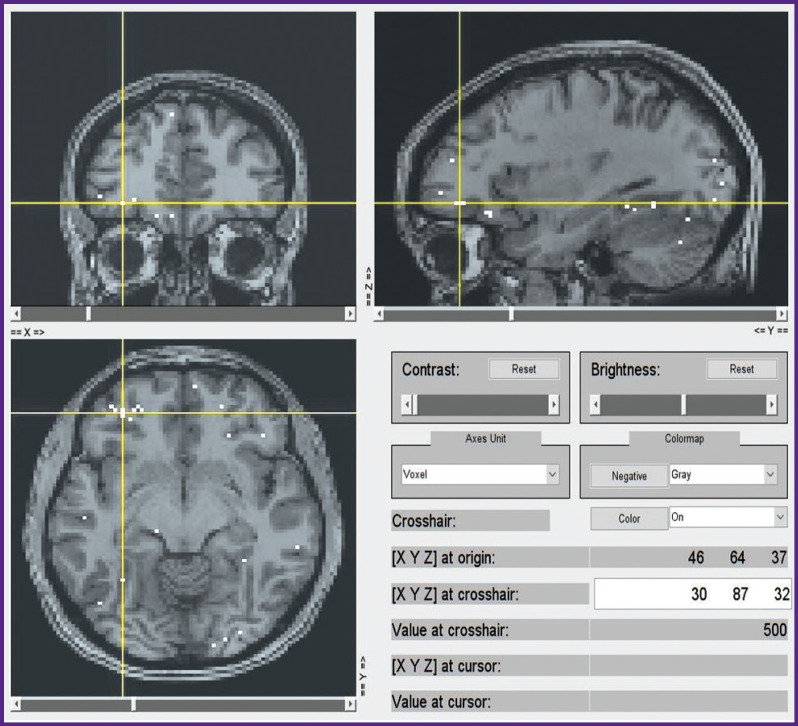
Localization of cluster *“Construction”*

A subsequent voxel-wise localization of these 15 semantic categories by modeling of fMRI data and by projecting them onto a conventional map of brain structures also seems to be sufficiently consistent. We currently computed this localization for the first 6 subjects out of 21, whereas only voxels with the highest weights were taken into account. A selection of group data from such an analysis is shown in Figures 10 to 13 for distribution of brain activity loci in the case of categories *“Conflict”*, *“Mental”*, *“Measure”*, and *“Construction”*, respectively.

**Figure 14:**
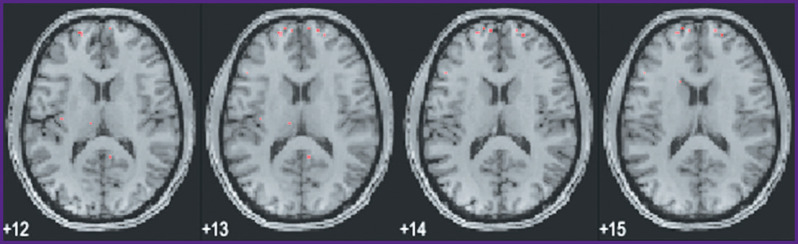
Brain activity which is semantically connected with the word *threat*, subject 1

**Figure 15:**
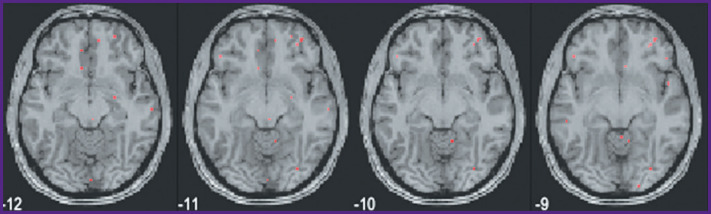
Brain activity which is semantically connected with the word *threat*, subject 2

Each subject demonstrated individual structure of feature words and *ipso facto* semantic representation in form of voxel activity of his/her brain. This is obvious in the case of the notion “threat”. Let us demonstrate the differences between the two subjects. In subject 1, the cluster, which is most close to threatening words, includes the following words from the initial stimulus material: *accusation, trust, respect, effort, victory, to free, duty*. The expertise showed that words of this cluster have semantics not so much of the threat but a successful overcoming of the threat. We have also to say that words such as *threat, to threat, war* that were present in the stimulus text did not get in the analysis of this subject. It means that for this subject these words do not lead to sufficient brain activation. At the same time, in subject 2 the closest cluster to the threatening words was the cluster, which included the words like *aggressive, to dominate, confrontation, invasion, enemy, to be sure, to threat*. Expert evaluation of this cluster confirms the presence of threat semantics. Differences in character of perception are also confirmed by the data from neurosemantic analysis. In both cases, we have observed activation of prefrontal brain. However, subject 1 shows bilateral activation of frontopolar zones (Figure 14), when subject 2 demonstrates activation of the right orbitofrontal field (Figure 15) more suitable for purely emotional processing [14].

## Discussion

As in several earlier studies [6], our results of the global comparison of subjects’ brain activation during listening to continuous segments of meaningful texts are rather disappointing. The improvement of registration technology by using ultrafast multiband fMRI protocol, as well as the use of more dramatic content in text composition (with elements of self-reference and overcoming of imminent threat) failed to reveal any consistency in the macroanalysis of semantic representations. The only significant contrast was found in activity of the amygdalae, which can be explained by the role of this double structure as a part of emotional network of the brain [15], perhaps due to a higher level of anxiety in social science students confronted with description of technical devices.

What are reasons for the repeated failure of the cognitive subtraction methodology? A criticism of cognitive subtraction is as old as the first chronometric experiments by Franciscus Donders. Cognitive subtraction methodology rests on the assumption of pure insertion, i.e. that there are no interactions among the cognitive components of a task. In cognitive neuroscience this more or less implicit assumption would only work with strongly modular architectures. But even with such highly uncommon architectures the recent discovery that the BOLD response has the character of a travelling wave makes the assumption untenable [16–18].

In view of these limitations of cognitive subtraction methodology, the second line of our research, which we called “microanalysis”, is of particular interest. It corresponds to the recent tendency of combining brain mapping with ontological studies [19, 20]. Our reconstruction of basic clusters of word-embedding semantics in spoken Russian language is a good example. The significance of this reconstruction may extend beyond the framework of a particular imaging study. Indeed, from the 15 anchoring categories of our analysis, 6 seem to be specific for Russian without any obvious counter-part among the 12 clusters found for English in the experiments of Huth et al. [5]. As these clusters have also been identified on the basis of brain activation pattern, one can speculate that our subjects are particularly sensitive to all issues related to state and government, to history, destiny and national values, to literature and art, and to gender differences whereas masculine component of this last category has a complex composition including aspects of medical care and spirituality. Even when some clusters in both languages seem to be similar, a closer examination shows differences in nuances. For instance, *“Conflict”* in our ontology is related to more large-scale military confrontations while in Huth and his colleagues’ classification its associated meaning is rather *“Street violence”*.

Of course, one has to consider these speculations with caution, as they reflect data of a limited group of subjects listening to a particular set of texts. To the best of our knowledge, our results on the brain mapping of semantic categories are the first such results for a language other than English, and understandably the results are loaded with limitations. Still, one can see some striking similarities in brain semantic representations of both these languages. Firstly, the representations are widely distributed. It may be not as apparent in English mapping, where the results are projected on an artificially constructed surface of the neocortex [5], but in a number of our categories one can clearly see that deep subcortical structures are involved, as is the case with categories such as *“Conflict”, “Mental”*, and *“Social”*. The first two of these semantic categories also demonstrate lateralization towards the right hemisphere. This is another similarity with the previous data on the English language semantic brain mapping.

There are several shortcomings in the current approaches to the microanalysis of the brain mapping of semantic representations. In particular, the word embedding-based approaches in computational linguistics treat each stimulus word independently and thus ignore the influence of context on language perception. New modelling efforts are directed at overcoming this limitation [21]. Next, there are problems with the arbitrariness of certain steps in reducing uncertainty in semantic mapping. As an option, we now consider replacement of the k-means procedure by the hierarchical agglomerative clustering. Finally, the multidimensional approaches to brain semantic mapping have been criticized for being logically circular: one incorporates regularities of language organization in the construction of the feature words vectors and then finds similar correlations in the brain semantic representations computed with the help of the feature words vectors. Like our North American colleagues [22], we believe that this alleged circularity cannot be avoided in ecologically valid studies, since the regularities of language (and the world that they reflect) shape the processing correlations in the brain.

This study demonstrated feasibility of our microstructural approach to semantic mapping. The new method allowed us to successfully localize brain mechanisms of semantic processing. It allowed us also to see the individual differences in perception of threat on the basis of relatively small texts. Of particular interests are voxel-wise date on brain activation, which can be for the first time compared with continuous stream of meaningful speech. One has to acknowledge finally that the very fact of discovered broad representation of semantic categories has earlier been noted in experimental works [23] and also predicted theoretically [24].

## Conclusion

This is the first report on semantic brain mapping based on the words-embedding approach to neurolinguistic computation in the Russian language. In comparison to the more traditional cognitive subtraction approach, the microanalysis seems to provide more stable and promising results, particularly with respect to the distribution of semantic categories across the human brain. One can see some striking similarities in brain semantic representations of both languages. Firstly, the representations are widely distributed. It may be not as apparent in English mapping, where the results are projected on an artificially constructed surface of the neocortex [5], but one can clearly see that deep subcortical structures are involved in a number of Russian-language categories such as *“Conflict”, “Mental”*, and *“Social”*. Some of these semantic categories also demonstrate lateralization towards the right hemisphere. This is another similarity with previous data on English-language semantic brain mapping. The significance of the latter finding is that it provides a further challenge to the established view on the left hemisphere’s monopoly in linguistic processing.

All the caveats notwithstanding, our final note and the main conclusion is that the methodology for brain imaging studies in neurolinguistics and in the emerging science of intersubjectivity is growing rapidly [20, 25]. At this early stage of research it would be premature to completely dismiss even the old-fashioned cognitive subtraction approach. Perhaps, in the future studies, both microanalysis and macroanalysis can be combined into a kind of meso-level approach. Relevant example is the temporal evolution of narratives with a pronounced self-referential content that changes the emotional perception of an initially dangerous situation after a resolution of the underlying conflict. In the case of such complex texts, contrasts of brain semantic representation before, during and after the conflict resolution would be of great scientific interest.

## Footnotes

1 Initially, 500 nouns and 500 verbs were selected but then the numbers were reduced by excluding obvious synonyms.

2 Release 016a from December 19, 2017 (courtesy Center for Magnetic Resonance Research, University of Minnesota).
